# ATG7 Promotes Bladder Cancer Invasion via Autophagy‐Mediated Increased ARHGDIB mRNA Stability

**DOI:** 10.1002/advs.201801927

**Published:** 2019-02-22

**Authors:** Junlan Zhu, Zhongxian Tian, Yang Li, Xiaohui Hua, Dongyun Zhang, Jingxia Li, Honglei Jin, Jiheng Xu, Wei Chen, Beifang Niu, Xue‐Ru Wu, Sergio Comincini, Haishan Huang, Chuanshu Huang

**Affiliations:** ^1^ Zhejiang Provincial Key Laboratory for Technology and Application of Model Organisms Key Laboratory of Laboratory Medicine Ministry of Education School of Laboratory Medicine and Life Sciences Wenzhou Medical University Wenzhou Zhejiang 325035 China; ^2^ Department of Environmental Medicine New York University School of Medicine New York NY 10010 USA; ^3^ Department of High‐Performance Computing Technology and Application Development Computer Network Information Center Chinese Academy of Sciences Beijing 100190 China; ^4^ Departments of Urology and Pathology New York University School of Medicine New York NY 10016 USA; ^5^ VA Medical Center in Manhattan New York NY 10010 USA; ^6^ Department of Biology and Biotechnology University of Pavia 27100 Pavia Italy

**Keywords:** ARHGDIB, ATG7, autophagy, bladder cancer, cancer invasion

## Abstract

Since invasive bladder cancer (BC) can progress to life threatening metastases, understanding the molecular mechanisms underlying BC invasion is crucial for potentially decreasing the mortality of this disease. Herein, it is discovered that autophagy‐related gene 7 (ATG7) is remarkably overexpressed in human invasive BC tissues. The knockdown of ATG7 in human BC cells dramatically inhibits cancer cell invasion, revealing that ATG7 is a key player in regulating BC invasion. Mechanistic studies indicate that MIR190A is responsible for ATG7 mRNA stability and protein overexpression by directly binding to ATG7 mRNA 3′‐UTR. Furthermore, ATG7‐mediated autophagy promotes HNRNPD (ARE/poly(U)‐binding/degradation factor 1) protein degradation, and in turn reduces HNRNPD interaction with ARHGDIB mRNA, resulting in the elevation of ARHGDIB mRNA stability, and subsequently leading to BC cell invasion. The identification of the MIR190A/ATG7 autophagic mechanism regulation of HNRNPD/ARHGDIB expression provides an important insight into understanding the nature of BC invasion and suggests that autophagy may represent a potential therapeutic strategy for the treatment of human BC patients.

## 1 Introduction

Macroautophagy/autophagy is a membrane trafficking and intracellular degradation system that delivers cytoplasmic elements to the lysosome.^1^ During this process, targeted dysfunctional or unnecessary cellular constituents are isolated in autophagosome. And then it fuses with a lysosome for further removal of the targeted cytoplasmic components.^2^ Autophagy degrades the damaged proteins and inhibits cell growth to exhibit its tumor suppression function.^3^ Alternatively, it plays tumor promoting roles through autophagy‐mediated intracellular recycling in some cancers, which provides substrates for metabolism and maintains the functional pool of mitochondria.^4^ Therefore, defining the mechanisms and the complex role for autophagy in cancer will be useful in guiding autophagy‐based therapeutic intervention.

ATG7 is an E‐1 enzyme and participates in activating ubiquitin‐like proteins (UBL) such as ATG12 and ATG8 for their transferring to an E‐2 enzyme, which is crucial for the autophagosome formation of the canonical pathway.^5^ It has been reported that withdrawal of nutrients triggers the induction of ATG7, which can bind to TP53, and then regulate the cell cycle inhibitor CDKN1A transcription, and eventually activate the pathway of cell death.^6^ Liver specific deletion of ATG7 has been reported as promoting the development of benign liver tumors,^7^ suggesting that autophagy acts as a tumor suppressor at the early stage of hepatocarcinogenesis. However, nothing is known about the potential contribution of ATG7 to cancer development at late stages, including cancer progression and invasion.

MicroRNAs (miRNAs) are short noncoding RNAs (≈22 nucleotides) that play essential roles in the regulation of numerous biological contexts.^8^ The tumor suppressive or oncogenic miRNAs could regulate either autophagic cell death or cytoprotective autophagy, both of which play the distinct roles in similar biological processes. For examples, MIR376B plays an oncogenic role by directly reducing BECN1 and ATG4C protein translation, and attenuates rapamycin‐induced autophagy in Huh‐7 and MCF‐7 cells,^9^ whereas MIR30A negatively regulates BECN1 expression and decreases autophagy activity in rapamycin‐treated T98G cells.^10^ Herein, we found that MIR190A promoted BC invasion and autophagy via stabilizing ATG7 mRNA by directly binding to its 3′‐untranslated region (UTR).

Bladder cancer (BC) is the fourth most lethal urological malignant tumor in men and fifth most common malignancy in the US.^11^ Survival of patients with muscle‐invasive bladder cancer (MIBC) is poor; the 5‐year survival rate for metastatic bladder cancer is only 6%.^12^ Thus, understanding the molecular mechanisms that trigger BC invasion and metastasis will be essential for clinical management. To the best of our knowledge, although multiple genetic and epigenetic factors have thought to be associated with uncontrolled BC invasion and metastatic spread,^13^ the potential contribution of autophagy response to BC invasion has never been explored. Here, for the first time, we demonstrated that ATG7 is overexpressed in human BC cell lines, mouse invasive BCs, and human BC tissues. ATG7 overexpression is important for the invasion behavior of human BC cells through its regulating autophagic removal of HNRNPD and consequent modulation of ARHGDIB mRNA stability, and BC invasion.

## 2 Results

### 2.1 ATG7 Protein was Remarkably Upregulated in Mouse Invasive BCs, Human BC Cell Lines, and Tissues

**Figure 1:**
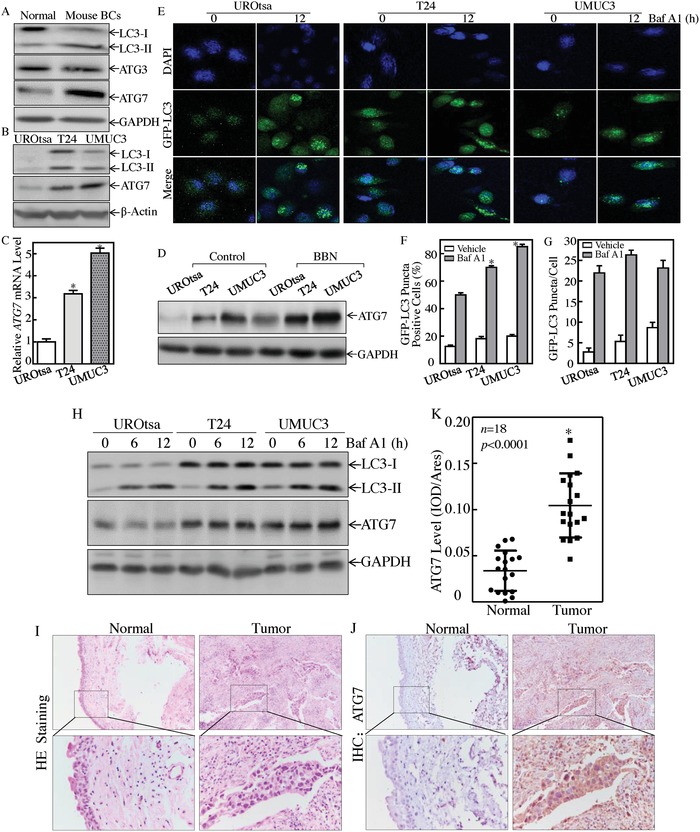
ATG7 was overexpressed in mouse invasive BCs, human invasive BC cells, and tissues. A) Western Blot assay was performed to detect the conversion of LC3 from LC3‐I to LC3‐II, ATG3 and ATG7 expression in mouse invasive BCs (*n* = 5). B) Western Blot was used to determine the conversion of LC3 from LC3‐I to LC3‐II and ATG7 protein expression, β‐Actin was used as a protein loading control. C) Real‐time PCR was performed to detect ATG7 mRNA expression, and the asterisk (*) indicates a significant increase from normal UROtsa cells (*p* < 0.05). D) UROtsa, T24, and UMUC3 cells were seeded into six‐well plates and the cells were then treated with or without 400 × 10^−6^ m of BBN for 24 h. The cell extracts were subjected to Western Blot for the determination of protein expression as indicated. GAPDH was used as a protein loading control. E) The GFP‐LC3 construct was stably transfected into UROtsa, T24, and UMUC3 cells, and then treated with 5 × 10^−9^ m Baf A1 for 12h. LC3 puncta formation was observed and images were captured using fluorescence microscopy. F,G) Percentage of GFP‐LC3 puncta cells (F) and the number of puncta per positive cell (G) were calculated. The asterisk (*) indicates a significant increase as comparison to UROtsa cells treated with Baf A1 (*p* < 0.05). H) Western Blot was performed to determine autophagy flux and ATG7 expression in presence of 5 × 10^−9^ m of Baf A1. I,J) Hematoxylin‐eosin (HE) and IHC staining were performed to evaluate morphology and ATG7 expression in 18 paired human BC tissues and their adjacent normal bladder tissues. The IHC images were captured using the AxioVision Rel.4.6 computerized image system. K) The ATG7 protein expression levels were analyzed by calculating the integrated IOD/area using Image‐Pro Plus version 6.0. Three independent experiments were performed, the Student's *t*‐test was utilized to determine the *p*‐value; and the asterisk (*) indicates a significant increase from the adjacent normal bladder tissues (*p < 0.05).

During our exploration of the molecular mechanisms underlying mouse invasive bladder carcinogenesis, a marked conversion of microtubule associated protein 1 light chain 3 (LC3) from LC3‐I to LC3‐II, upregulation of autophagy‐related protein ATG7 was unexpectedly observed in mouse invasive BCs (*n* = 5) (**Figure** **1**A), revealing the potential association of autophagy with BC invasion. Moreover, the conversion of LC3 from LC3‐I to LC3‐II, the expression of ATG7 protein and mRNA in both human invasive BC cells T24 and UMUC3, was much higher than those observed in normal human urothelial cell UROtsa (Figure 1B,C). Moreover, treatment of cells with *N*‐butyl‐*N*‐(4‐hydroxybutyl)nitrosamine (BBN), a genotoxic bladder carcinogen, promoted ATG7 expression in all three cell lines, including UROtsa, T24, and UMUC3 (Figure 1D). To further determine the autophagy activity in UROtsa, T24, and UMUC3 cells, we stably transfected with a GFP‐LC3 plasmid into these three cells. Following the treatment with Bafilomycin A1 (Baf A1) for 12 h, the percentage of GFP‐LC3 puncta‐positive cells was much higher in T24 and UMUC3 cells in comparison to UROtsa cells, although average number of puncta in each cells were comparable among three cells (Figure 1E–G). To investigate the potential association of the autophagic flux in human BC cell invasion, we employed autophagy inhibitor Baf A1 to evaluate autophagic status in T24, UMUC3, and UROtsa cells. As shown in Figure 1H, the autophagic flux in UMUC3 and T24 cells was significantly higher than in UROtsa cells. It is worth noting that expression of ATG7 protein was also consistently elevated in these invasive BC cells in comparison to those in normal urothelial cell line UROtsa. Most importantly, the results obtained from analyses of ATG7 expression in 18 paired human BC tissues and their adjacent normal bladder tissues indicated that ATG7 was upregulated in human BCs (Figure 1I–K). Collectively, our results strongly demonstrate that autophagy and ATG7 protein expression are elevated in human invasive BC cells in vitro, mouse invasive BC tissues, and human BC tissues in vivo.

### 2.2 ATG7 Overexpression Attributed to Upregulated MIR190A‐Mediated Stabilization of ATG7 mRNA

**Figure 2:**
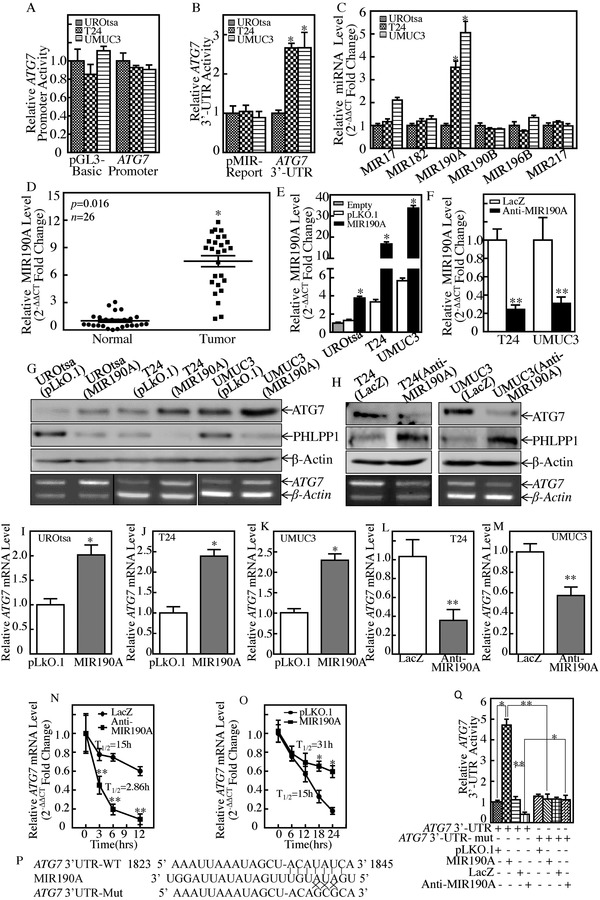
MIR190A stabilized ATG7 mRNA by direct binding to 3′‐UTR of ATG7 mRNA in human bladder cancer cells. A,B) The pGL3‐Basic vector versus human ATG7 promoter‐driven luciferase reporter (A) or pMIR‐report versus pMIR‐ATG7 3′‐UTR reporter (B) were transiently transfected into the indicated cells and luciferase activity was evaluated. Student's *t*‐test was utilized to calculate the *p*‐value, *p* > 0.05 (A), and *p < 0.05 (B). C) qPCR was performed to determine the effect of miRNA expression in the indicated cells (*p < 0.05). D) Quantitative Real‐time PCR analyses were used to determine MIR190A expression in human cancerous (T) and paired normal (N) tissues among 26 bladder cancer patients. Student's *t*‐test was utilized to determine the *p*‐value, *p = 0.016. E,F) UROtsa, T24, and UMUC3 cells were stably transfected with MIR190A constitutively expressed plasmids (E), while MIR190A antisense plasmids were stably transfected into T24 and UMUC3 cells (F). The asterisk (*) indicates a significant increase in comparison to UROtsa empty cells (*p < 0.05) (E), while the double asterisk (**) indicates a significant decrease in comparison to scramble vector control (**p < 0.05) (F). G,H) Cell lysates and total RNAs extracted from the indicated cells were subjected to either Western Blot (top panel) to determine ATG7 and PHLPP1 protein expression or RT‐PCR (bottom panel) to determine ATG7 mRNA expression, respectively. β‐Actin was used as a loading control. I–M) The ATG7 mRNA levels of indicated cells were evaluated by real‐time PCR (*p < 0.05, **p < 0.05). N) In the presence of Actinomycin D (Act D), ATG7 mRNA degradation rate in the indicated cell transfectants was determined for the indicated time, **p < 0.05. O) UMUC3(MIR190A) cells and its vector control transfectants were used to test the ATG7 mRNA degradation as described in (N), *p < 0.05. P) Schematic of the construction of the ATG7 mRNA 3′‐UTR luciferase reporter and its mutants was aligned with MIR190A. Q) Wild‐type and mutant of ATG7 3′‐UTR luciferase reporters were transiently cotransfected with pRL‐TK into the indicated cells. ATG7 3′‐UTR activity of each transfectant was determined (*p < 0.05, **p < 0.05).

MiRNAs are able to bind to the 3′‐untranslated region of target gene mRNA and affect the stability or translation of their targeted mRNAs which regulate diverse biological processes such as cell growth, metastasis, and tumorigenesis.^14^ Based on the results above, which show consistent elevation of both ATG7 protein and mRNA in high grade human BC cell lines, we then detected whether ATG7 mRNA was upregulated at either transcription level or mRNA stability. The results from the determination of mRNA transcription using ATG7 promoter‐driven luciferase reporter showed no significant difference between UROtsa, T24, and UMUC3 cells (**Figure** **2**A). Therefore, the possibility of transcriptional regulation was excluded. And next, the potential difference of ATG7 mRNA 3′‐UTR activity was evaluated among the three cell lines. The results showed that ATG7 mRNA 3′‐UTR activity in high grade T24 and UMUC3 cells was significantly higher than that observed in UROtsa cells (Figure 2B), revealing that miRNAs might be involved. To test this notion, TargetScan (v7.0; targetscan.org),^15^ PicTar (pictar.org),^16^ and miRanda (microrna.org)^17^ were used to search the putative miRNAs. The results indicated that there were multiple putative miRNA binding sites in 3′‐UTR of ATG7 mRNA, including binding sites for MIR17, MIR182, MIR190A, MIR190B, MIR196B, and MIR217 (Figure S1A, Supporting Information). The differential expression of the above miRNAs was evaluated among UROtsa, T24, and UMUC3 cells. As shown in Figure 2C, MIR190A was identified to be upregulated in T24 and UMUC3 cells in comparison to UROtsa cells. To extend our finding to in vivo human BCs, we compared MIR190A expression between human BC tissues (*n* = 26) and their adjacent normal bladder tissues. The results showed that MIR190A expression was remarkably increased in human BC tissues in comparison to their normal counterparts (Figure 2D). To identify the effect of MIR190A, a construct expressing MIR190A was transfected into UROtsa, T24, and UMUC3 cells, respectively. The stable transfectants named UROtsa(MIR190A), UMUC3(MIR190A), and T24(MIR190A) were identified (Figure 2E). Ectopic expression of MIR190A resulted in downregulation of PHLPP1 expression (known target of MIR190A) and remarkable upregulation of ATG7 protein in UROtsa, T24, and UMUC3 cells (Figure 2G) as compared to scramble vector transfectants. In contrast to MIR190A ectopic expression, deficiency of MIR190A expression by stable transfection of MIR190A antisense (Figure 2F) increased PHLPP1 protein expression and impaired ATG7 protein expression (Figure 2H). The results obtained from real‐time polymerase chain reaction (PCR) also showed a consistent modulation of ATG7 upon MIR190 overexpression or suppression (Figure 2I–M). Our results clearly reveal that MIR190A mediates ATG7 expression. To illuminate the mechanisms, the effect of MIR190A antisense on ATG7 mRNA stability was evaluated in UMUC3(Anti‐MIR190A) and scramble transfectant UMUC3(LacZ). As shown in Figure 2N, MIR190A‐antisense promoted ATG7 mRNA degradation, while the ectopic expression of MIR190A stabilized ATG7 mRNA (Figure 2O), suggesting that MIR190A is an essential regulator of ATG7 mRNA stabilization. Then, the MIR190A binding site in ATG7 3′‐UTR‐luciferase reporter was mutated and constructed (Figure 2P). The mutated and wild‐type ATG7 3′‐UTR luciferase reporter was cotransfected with pRL‐TK transiently into either UMUC3(MIR190A), UMUC3(Anti‐MIR190A), or their vector scramble transfectants. The results showed that ATG7 3′‐UTR luciferase activity was significantly increased in UMUC3(MIR190A) cells as compared with that in UMUC3(pLKO.1), and profoundly inhibited in UMUC3(Anti‐MIR190A) cells as compared with that in UMUC3(LacZ) transfectant, whereas the MIR190A binding site mutation in ATG7 3′‐UTR luciferase reporter attenuated the responses of UMUC3 cells to ectopic expression of either MIR190A or Anti‐MIR190A (Figure 2Q). These results strongly demonstrate that MIR190A increases ATG7 mRNA stabilization by directly binding to its 3′‐UTR.

### 2.3 ATG7 and its Mediated Autophagy were Essential for Human BC Invasion

**Figure 3:**
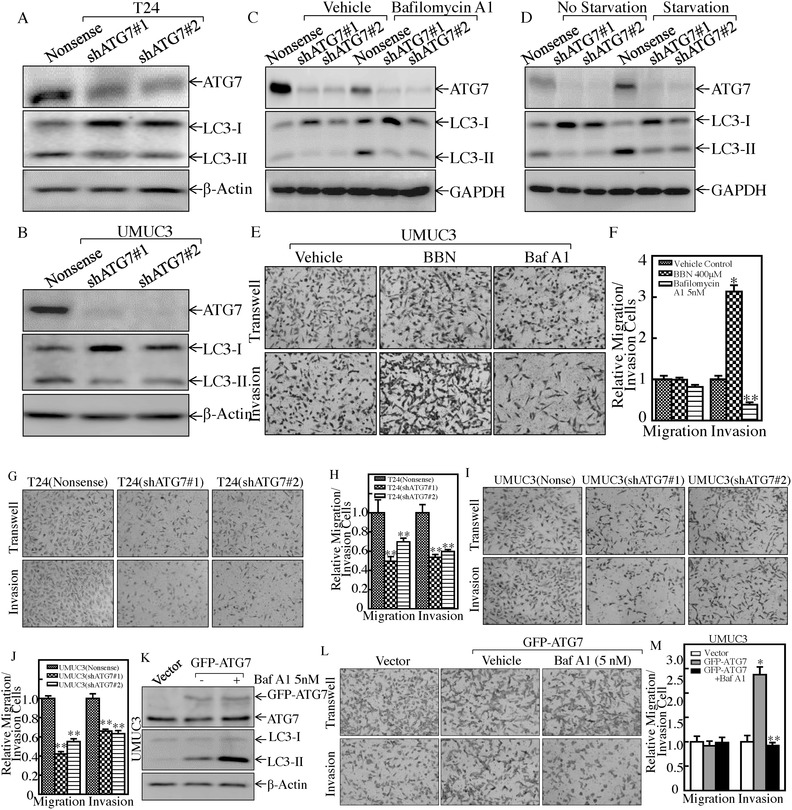
ATG7 overexpression and its mediated autophagy were crucial for BC invasion. A,B) Knockdown constructs of ATG7 were stably transfected into T24 and UMUC3 cells, respectively. Western Blot was used to detect the knockdown efficiency of ATG7 protein and autophagy activity. C) The indicated cells were treated with or without Baf A1 for 24 h, and the cell extracts were subjected to Western Blot to determine protein expression, as indicated. D) The indicated cells were cultured in either 10% fetal bovine serum (FBS) medium (no starvation) or 0.1% FBS medium (starvation) for 24 h. The cell extracts were subjected to Western Blot to determine protein expression, as indicated. E,F) The invasive abilities of UMUC3 cells were determined in presence of 400 × 10^−6^ m of BBN or 5 × 10^−9^ m of Baf A1 using BD BioCoat Matrigel Invasion Chamber applied with the matrigel. After incubation for 24 h, the cells were fixed and stained as described in the Experimental Section. The migrated and invasive cells were photographed under an Olympus DP71 (E), and the number of the cells in each image was counted by the software “Image J.” The invasion rate was normalized with the insert control according to the manufacturer's instruction (F). The bars represent mean ± SD from three independent experiments. Student's *t*‐test was utilized to determine the *p*‐value; the asterisk (*) indicates a significant increase in comparison to vehicle control (*p < 0.05) (E), while the double asterisk (**) indicates a significant decrease in comparison to vehicle control (**p < 0.05). G–J) The invasion abilities of T24(shATG7), UMUC3(shATG7), and their nonsense transfectants were determined, **p < 0.05. K) UMUC3(Vector) and UMUC3(GFP‐ATG7) were treated with 5 × 10^−9^ m of Baf A1 for 12 h, and the cell extracts were subjected to Western Blot for determination of ATG7 overexpression and autophagic status. β‐Actin was used as a protein loading control. L,M) The invasion abilities of the indicated cells were determined in presence or absence of 5 × 10^−9^ m of Baf A1. The asterisk (*) indicates a significant increase in comparison to vector control transfectant (*p < 0.05), while the double asterisk (**) indicates a significant inhibition in comparison to vehicle control cells (**p < 0.05).

To understand the association between overexpressed ATG7 with elevated autophagy in highly invasive BC cells, small hairpin RNA specifically targeting human ATG7 (shATG7) was used to knock down ATG7 in T24 and UMUC3 cells, and the effect of LC3‐I conversion into lipidated LC3‐II was evaluated, as shown in **Figure** **3**A,B. The results showed that the deficiency of ATG7 expression by its short hairpin RNA (shRNA) led to a significant inhibition of autophagy activity in both T24 and UMUC3 cells. This notion was further supported by the observation of the effect of autophagy inhibitor Baf A1 and the starvation on ATG7 expression and autophagy (Figure 3C,D). To test the association of autophagy with BC invasion, we assessed the effect of BBN and Baf A on the invasive abilities of UMUC3 cells. The results showed that BBN significantly promoted the invasive abilities of UMUC3 cells, while inhibition of autophagy by Baf A1 exhibited inhibitory effect on invasion of UMUC3 cells (Figure 3E,F). Consistently, the deficiency of autophagy in ATG7 knockdown transfectants also showed inhibition of the invasive abilities in both T24 and UMUC3 cells (Figure 3G–J), while the deficiency of BECN1 promoted the invasive abilities in UMUC3 cells (Figure S1B–D, Supporting Information), suggesting that ATG7 plays a critical role in human invasive BC invasion. To explore whether ATG7‐autophagic flux directly mediates human BC invasion, we stably transfected GFP‐tagged ATG7 overexpression construct into UMUC3 cells. The stable transfectants UMUC3(GFP‐ATG7) and UMUC3(Vector) were used to evaluate their invasive abilities in presence or absence of autophagy inhibitor Baf A1. The results showed that ATG7 overexpression resulted in a remarkable increase in autophagy and BC invasion in comparison to its vector control transfectant, whereas treatment of cells with Baf A1 attenuated the autophagy induction accompanied with reduction of BC invasion due to ATG7 overexpression (Figure 3K–M). These results reveal that ATG7‐mediated autophagy is at least one of key events for ATG7 promotion of BC invasion.

**Figure 4:**
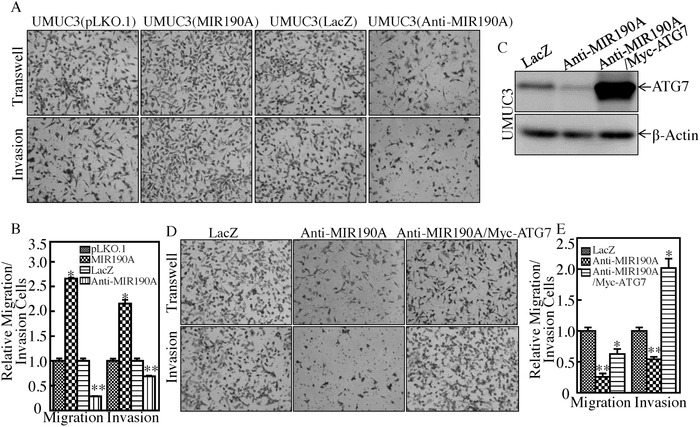
ATG7 was an MIR190A downstream effector responsible for BC invasion. A,B) The invasion abilities of the indicated cells were evaluated. Student's *t*‐test was utilized to determine the *p*‐value; the asterisk (*) indicates a significant increase in comparison to UMUC3(pLKO.1) transfectants (*p < 0.05) (B), while the double asterisk (**) indicates a significant decrease in comparison to UMUC3(LacZ) transfectants (**p < 0.05). C) ATG7 overexpressed plasmid was stably transfected into UMUC3(Anti‐MIR190A) cells. The overexpression efficiency of ATG7 protein was assessed by Western Blotting. β‐Actin was used as an internal protein loading control. D) The invasion abilities of UMUC3(Anti‐MIR190A) and UMUC3(Anti‐MIR190A/ATG7) cells were evaluated. Student's *t*‐test was utilized to determine the *p*‐value; the double asterisk (**) indicates a significant decrease in comparison to scramble vector transfectants (**p < 0.05), while the asterisk (*) indicates a significant increase in comparison to Anti‐MIR190A transfectants (*p < 0.05) (E).

The above results reveal that ATG7 overexpression promotes human BC invasion, and that MIR190A stabilizes ATG7 mRNA by directly binding to its 3′‐UTR. We next detected the effect of MIR190A on human bladder cancer invasion. The results indicated that the ectopic expression of MIR190A remarkably promoted invasion of UMUC3 cells (**Figure** **4**A,B), while introduction of control MIR196B in UMUC3 cells did not exhibit an effect on the invasion of UMUC3 cells (Figure S2A–C, Supporting Information). Moreover, the inhibition of MIR190A expression attenuated the invasive abilities of UMUC3 cells (Figure 4A,B). To evaluate the role of ATG7 in MIR190A mediation of BC invasion, we stably transfected ATG7 constitutively expressed plasmid into UMUC3(Anti‐MIR190A) cells to see whether ectopic expression of ATG7 could reverse the inhibitory effect of Anti‐MIR190A on BC invasion. We found that the inhibition of Anti‐MIR190A on BC invasion was completely reversed with introduction of ATG7 in UMUC3(Anti‐MIR190A) cells (Figure 4C–E). These results reveal that ATG7 is an MIR190A downstream effector responsible for MIR190A promotion of BC invasion.

### 2.4 ARHGDIB was an ATG7 Downstream Effector for Bladder Cancer Cell Invasion

**Figure 5:**
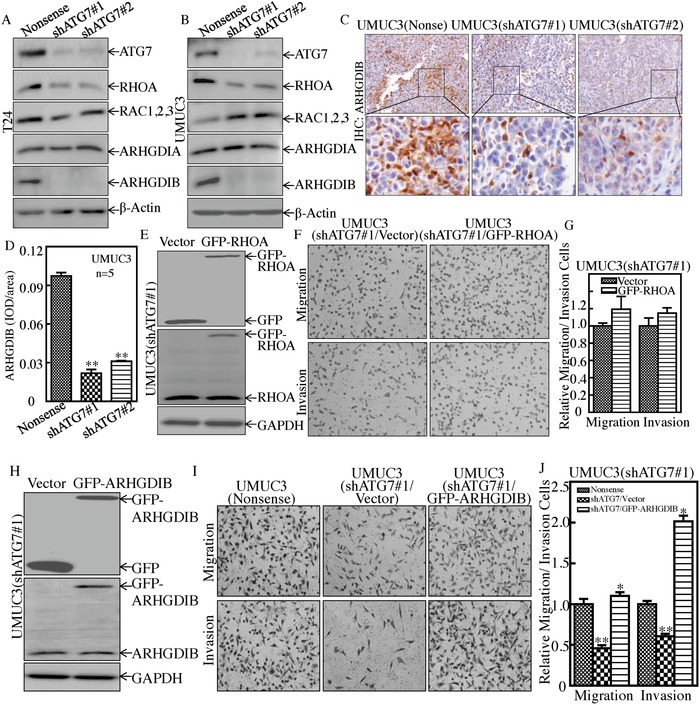
ARHGDIB, but not RHOA, mediated ATG7 promotion of BC invasion. A,B) Western Blot was used to determine protein expression of ATG7, RHOA, RAC1,2,3, ARHGDIA, and ARHGDIB. β‐Actin was used as a protein loading control. C,D) Athymic nude mice were injected with UMUC3(Nonsense) cells (*n* = 5), UMUC3(shATG7#1) cells (*n* = 5), or UMUC3(shATG7#2) cells (*n* = 5), respectively. IHC staining was performed to evaluate ARHGDIB expression. The IHC images were captured using the AxioVision Rel.4.6 computerized image system and protein expression levels were analyzed by calculating the integrated IOD/area using Image‐Pro Plus version 6.0. Results are presented as the mean ± SD of five mice in each group. Student's *t*‐test was utilized to determine the *p*‐value, **p < 0.05. E) GFP‐RHOA expression constructs were stably transfected into UMUC3(shATG7#1) cells, and the stable transfectants were identified by Western Blotting. F,G) The invasion abilities of UMUC3(shATG7#1/Vector) cells and UMUC3(shATG7#1/GFP‐RHOA) cells were determined, as described in the Experimental Section. Results are presented as the mean ± SD from triplicate (*p* > 0.05). H) GFP‐ARHGDIB expression constructs were stably transfected into UMUC3(shATG7#1) cells and the stable transfectants were identified by Western Blotting. I,J) The invasion abilities of UMUC3(Nonsense), UMUC3(shATG7#1/Vector), and UMUC3(shATG7#1/GFP‐ARHGDIB) cells were determined, as described in the Experimental Section. Bars represent mean ± SD from three independent experiments. Student's *t*‐test was utilized to determine the *p*‐value. The double asterisk (**) indicates a significant decrease in comparison to scramble vector transfectants (**p < 0.05), while the asterisk (*) indicates a significant increase in comparison to UMUC3(shATG7#1/Vector) transfectants (*p < 0.05) (J).

ARHGDIA, ARHGDIB, RAC1,2,3, and ras homolog gene family, member A (RHOA) are key regulators of actin polymerization, cell migration, and cancer invasion.^18^ To clarify the mechanism underlying the ATG7 regulation of BC invasion, ARHGDIA, ARHGDIB, RAC1,2,3, and RHOA proteins were determined. As shown in **Figure** **5**A,B, knockdown of ATG7 resulted in a dramatic attenuation of ARHGDIB and RHOA protein in T24 and UMUC3 cells, while did not show consistent impact on ARHGDIA and RAC1,2,3 protein expression. Further, the inhibitory effect of ATG7 on ARHGDIB expression was also observed in in vivo xenograft nude mice injected with the ATG7 knockdown BC cells (Figure 5C,D). Thus, we next stably transfected GFP‐RHOA into UMUC3(shATG7#1) transfectant (Figure 5E). Overexpression of GFP‐RHOA in UMUC3(shATG7) cells did not show a reversible effect on BC invasion (Figure 5F,G), excluding the potential contribution of RHOA in ATG7‐mediated BC invasion. We next transfected ectopic expression of GFP‐ARHGDIB into UMUC3(shATG7#1) cells (Figure 5H) and its effects on cell invasion were detected. As shown in Figure 5I,J, overexpression of GFP‐ARHGDIB reversed the deficiency of invasion in UMUC3(shATG7#1) cells. Our results demonstrate that ARHGDIB is at least an ATG7 downstream effector for promotion of invasion of BCs.

### 2.5 ATG7 Stabilized ARHGDIB mRNA through Attenuating HNRNPD Protein Expression

**Figure 6:**
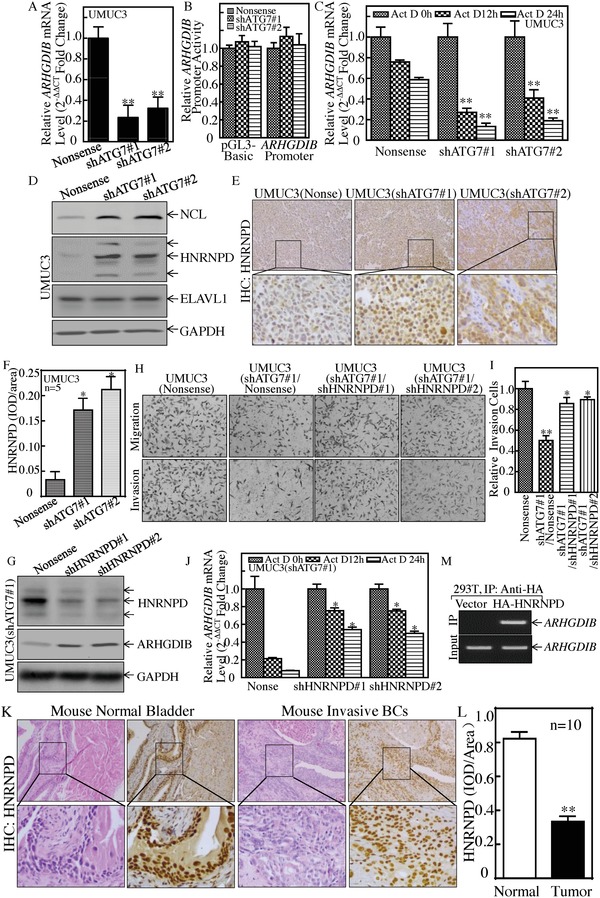
HNRNPD was an ATG7 downstream effector and inhibited ARHGDIB mRNA stability and BC cell invasion. A) Real‐time PCR was used to determine ARHGDIB mRNA expression, and β‐Actin was used as an internal control, **p < 0.05. B) Human ARHGDIB promoter‐driven luciferase activity was evaluated in the indicated cells, *p* > 0.05. C) ARHGDIB mRNA stability was detected in the presence of Act D by using real‐time PCR, **p < 0.05. D) The indicated cell extracts were subjected to Western Blot for determination of NCL, ELAVL1, and HNRNPD protein expression. E,F) IHC staining was performed to detect HNRNPD expression in the tumor tissues obtained from the nude mice injected with UMUC3(shATG7#1), UMUC3(shATG7#2), or UMUC3(Nonsense) cells, *p < 0.05. G–I) HNRNPD knockdown constructs were stably transfected into UMUC3(shATG7#1). The HNRNPD knockdown efficiency was evaluated by Western Blotting (G). The stable transfectants were used to determine their invasion abilities in comparison to nonsense control transfectants (H and I). Student's *t*‐test was utilized to determine the *p*‐value, the double asterisk (**) indicates a significant decrease in comparison to scramble vector transfectants (**p < 0.05), while the asterisk (*) indicates a significant increase in comparison to UMUC3(shATG7#1/Nonsense) transfectants (*p < 0.05) (I). J) ARHGDIB mRNA stability was evaluated by real‐time PCR in the presence of Act D in UMUC3(shATG7#1/shHNRNPD) cells and its nonsense control, *p < 0.05. K,L) IHC staining was performed to evaluate HNRNPD expression in mouse highly invasive BCs, **p < 0.05. M) HA‐HNRNPD construct was transfected into 293T cells and HA‐HNRNPD protein was pulled down with anti‐HA beads. The mRNAs bound to HNRNPD protein were used to carry out RT‐PCR for determination of ARHGDIB mRNA expression.

To illuminate the mechanisms underlying ATG7's promotion of ARHGDIB protein expression, their mRNA levels were first examined. We found that ARHGDIB mRNA were profoundly impaired in ATG7 knockdown transfectants (**Figure** **6**A). The ARHGDIB promoter‐driven luciferase reporter and PGL3 basic luciferase reporter were next transfected into UMUC3(shATG7) cells to assess the potential ATG7 upregulation of ARHGDIB mRNA transcription, and it was found that ARHGDIB promoter transcriptional activity was comparable (Figure 6B), excluding the possibility that ATG7 upregulation of ARHGDIB mRNA transcription. Therefore, we explored the possibility that ATG7 stabilizes ARHGDIB mRNA. Upon treating with Act D, ARHGDIB mRNA degradated more faster in UMUC3(shATG7) than in UMUC3(Nonsense) cells (Figure 6C), consistent with these observations of ATG7 stabilization of ARHGDIB mRNA. The inhibition of MIR190A by Anti‐MIR190A promoted ARHGDIB mRNA degradation, thereby leading to a reduction of ARHGDIB mRNA expression (Figure S3A–C, Supporting Information). Our results reveal that ATG7 expression plays an essential role in maintaining ARHGDIB mRNA stability.

To determine the upstream regulators mediating ARHGDIB mRNA stabilization by ATG7, HNRNPD, ELAVL1, and nucleolin (NCL) (which are able to bind to many ARE‐mRNAs and regulate mRNA stability) were tested and compared. The data showed that NCL and HNRNPD were elevated, while ELAVL1 protein was comparable in ATG7 knockdown cells as compared to nonsense cells (Figure 6D). These results exclude the role of ELAVL1 and NCL on ATG7 stabilization of ARHGDIB mRNA. Consistent with the ATG7 negative regulation of HNRNPD in vitro cultured UMUC3 cells, HNRNPD protein expression was markedly increased in xenograft tumor tissues attained from nude mice injected with UMUC3(shATG7) cells (Figure 6E,F), and the similar change of NCL protein expression (Figure S3D,E, Supporting Information). Thus, we knocked down HNRNPD in T24 cells by using shRNAs specifically targeting human HNRNPD, and the stable transfectants, T24(shHNRNPD#1) and T24(shHNRNPD#2) and its scramble transfectant T24(Nonsense), were established and identified (Figure S4A, Supporting Information). As expected, HNRNPD knockdown in T24 cells led to increased expression of ARHGDIB protein as well as promotion of BC cell invasion (Figure S4B,C, Supporting Information). Further, we transfected shRNAs specifically targeting human HNRNPD into UMUC3(shATG7#1) cells, as shown in Figure 6G. The inhibition of HNRNPD expression in UMUC3(shATG7#1) restored ARHGDIB mRNA stability and its protein expression, as well as BC cell invasion (Figure 6H–J), suggesting that HNRNPD might be a mediator linking ATG7 to ARHGDIB and associated with BC invasion.

To evaluate the HNRNPD in in vivo, relevant to highly invasive bladder cancer carcinogenesis, HNRNPD expression was detected in mouse invasive bladder cancer tissues. The results obtained from the IHC staining with anti‐HNRNPD antibodies showed that HNRNPD protein was downregulated in the mouse invasive bladder tissues in comparison to normal bladder tissues (Figure 6K,L). To determine whether HNRNPD is able to directly bind with ARHGDIB mRNA, RNA‐IP assay was performed in HEK 293T cells that expressed HA‐HNRNPD. The results showed that HNRNPD did bind to ARHGDIB mRNA (Figure 6M). Taken together, our results validate that ATG7 inhibits HNRNPD expression, which results in less HNRNPD binding to ARHGDIB mRNA, consequently leading to ARHGDIB mRNA stabilization, and in turn promoting BC invasion.

### 2.6 ATG7 Overexpression Promoted Autophagic Removal of HNRNPD Protein in BC Cells

**Figure 7:**
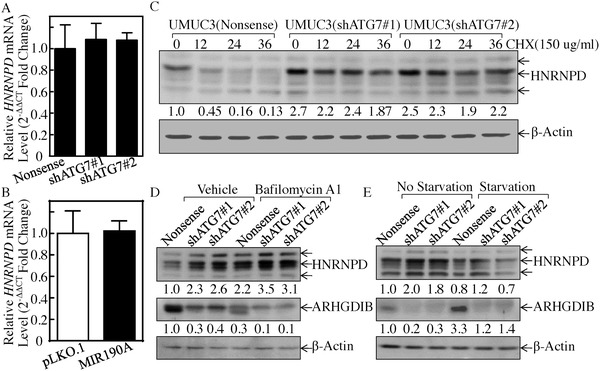
ATG7 promoted autophagic removal of HNRNPD protein. A,B) Real‐time PCR was used to determine HNRNPD mRNA expression in the indicated cells, *p* > 0.05. C) HNRNPD protein stability was evaluated in the presence of Cycloheximide (CHX) in the indicated cells. D) The indicated cells were treated with or without Baf A1 for 24 h, and the cell extracts were subjected to Western Blot to determine protein expression. E) The indicated cells were cultured in either 10% FBS medium (no starvation) or 0.1% FBS medium (starvation) for 24 h. The cell extracts were subjected to Western Blot to determine protein expression.

To elucidate how ATG7 inhibits HNRNPD protein expression, we assessed the effect of ATG7 on HNRNPD mRNA abundance in UMUC3 cells. As shown in **Figure** **7**A,B, HNRNPD mRNA's level was comparable between ATG7 knockdown transfectants and its scramble nonsense transfectants, MIR190A transfectants, and its pLKO.1 control. We next tested to see whether ATG7 regulated HNRNPD protein stability. The result indicated that ATG7 knockdown remarkably increased HNRNPD protein stability (Figure 7C). Consistently, ectopic expression of MIR190A also promoted HNRNPD protein degradation (Figure S4D, Supporting Information). Intriguingly, ATG7 plays an essential role in the process of autophagy, which delivers some proteins to lysosomes for degradation.^19^ Thus, we determined whether ATG7 overexpression‐mediated autophagy subsequently promoted HNRNPD protein degradation. We found that HNRNPD was markedly upregulated after inhibition of autophagy by Baf A1 treatment in comparison to the vehicle control (Figure 7D). Consistent with the alteration of HNRNPD protein, ARHGDIB was dramatically decreased (Figure 7D). However, starvation‐induced autophagy inhibited HNRNPD expression and elevated ARHGDIB expression (Figure 7E).

### 2.7 MIR190A Upregulation Mediated the Elevation of ATG7 Expression, HNRNPD Degradation, and ARHGDIB Overexpression

**Figure 8:**
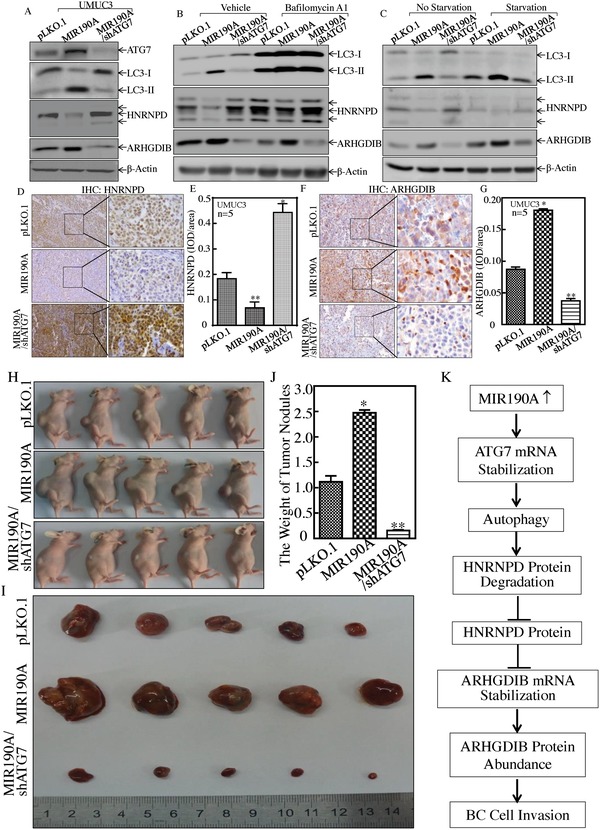
MIR190A upregulation mediated ATG7 elevation, HNRNPD degradation, and ARHGDIB overexpression. A) UMUC3(pLKO.1), UMUC3(MIR190A), and UMUC3(MIR190A/shATG7) cell extracts were subjected to Western Blot to evaluate the expression of ATG7, LC3, HNRNPD, and ARHGDIB. GAPDH was used as a protein loading control. B) UMUC3(pLKO.1), UMUC3(MIR190A), and UMUC3(MIR190A/shATG7) cells were treated with or without Baf A1 for 24 h, and the cell extracts were subjected to Western Blot to determine protein expression, as indicated. C) UMUC3(pLKO.1), UMUC3(MIR190A), and UMUC3(MIR190A/shATG7) cells were cultured in either 10% FBS medium (no starvation) or 0.1% FBS medium (starvation) for 24 h. The cell extracts were then subjected to Western Blot to determine protein expression, as indicated. D–G) The xenograft tumor tissues obtained from athymic nude mice injected with UMUC3(pLKO.1) cells (*n* = 5), UMUC3(MIR190A) cells (*n* = 5), or UMUC3(MIR190A/shATG7) cells (*n* = 5) were subjected to IHC for evaluation of the expression of HNRNPD (D,E) and ARHGDIB (F,G), *p < 0.05,**p < 0.05. H,I) Athymic nude mice were subcutaneously injected into the right axillary region of each mouse with UMUC3(pLKO.1), UMUC3(MIR190A), and UMUC3(MIR190A/shATG7) transfectants (2 × 10^6^ suspended in 100 µL PBS), as indicated in the Experimental Section. Four weeks after cell injection, the mice were sacrificed and the tumors were surgically removed and photographed (H,I), as well as weighed (J), *p* < 0.05, **p < 0.05. K) The proposed mechanisms underlying ATG7 overexpression in the promotion of human bladder cancer cells invasion: MIR190A upregulation promotes ATG7 overexpression, which further mediates the autophagic removal of HNRNPD, in turn increasing in ARHGDIB mRNA stability and protein expression, as well as BC invasion.

The above results clearly demonstrate that MIR190A stabilizes ATG7 mRNA through its direct binding to ATG7 mRNA 3′‐UTR in human BCs, and that MIR190A is crucial for ATG7‐induced autophagy and human BC invasion. To evaluate the contribution of ATG7‐mediated autophagy to MIR190A mediation of HNRNPD and ARHGDIB expression in BCs, we knocked down ATG7 in UMUC3(MIR190A) cells, as shown in **Figure** **8**A. The effect of the inhibition of autophagy by Baf A1 or induction of autophagy upon cell starvation on the expression of HNRNPD and ARHGDIB was evaluated in UMUC3(MIR190A) and UMUC3(MIR190A/shATG7) cells. As shown in Figure 8B, inhibition of autophagy by Baf A1 resulted in restoration of HNRNPD expression and reduction of ARHGDIB due to ectopic expression of MIR190A, in comparison to treatment of cells with vehicle only. Consistently, induction of autophagy by cell starvation showed an opposite effect on both protein expressions (Figure 8C). Moreover, HNRNPD and ARHGDIB expressions were consistently observed in in vivo tumors obtained from xenograft nude mice injected with UMUC3(vector), UMUC3(MIR190A), and UMUC3(MIR190A/shATG7) cells (Figure 8D–G). In addition, it is highly significant to note that xenograft tumor formation and growth, as demonstrated in tumor size and tumor weight, also showed that ectopic expression of MIR190A promoted tumor formation and growth, while knockdown of ATG7 in UMUC3(MIR190A) cells completely abolished the promotive effect of MIR190A on xenograft tumor growth (Figure 8H–J). Taken together, our findings reveal that MIR190A upregulation mediates ATG7 overexpression, which is crucial for the autophagic removal of HNRNPD, in turn promoting ARHGDIB mRNA stability and protein expression, as well as BC invasion both in vitro and in vivo as diagrammed in Figure 8K.

## 3 Discussion

In a wide variety of human cancers, impairments of autophagy physiological activation, assembly, and function pathway have been increasingly observed,^20^ but the exact role of autophagy in cancer initiation, growth, and progression depends on contextual demands, based on tissue/tumor types. Invasive bladder cancer is one of the most common types of cancer and exists as a major therapeutic challenge.^21^ The current studies focus on the status and functions of ATG7 expression and its mediated autophagy in human BCs. We have also identified the ATG7 upstream regulator leading to ATG7 overexpression as well as elucidated the ATG7 downstream role effecting on BC invasion. We have discovered that ATG7 expression is markedly overexpressed in mouse invasive BCs, human BC cell lines, and human BC tissues. Mechanistic reveals that MIR190A is a key upstream regulator responsible for the stabilization of ATG7 mRNA. The biological role of overexpressed ATG7 is demonstrated by knockdown of ATG7 in human BC cells, which show a dramatical inhibition of BC cancer cell invasion. Our results also indicate that ATG7 overexpression promotes autophagy responses, consequently removing HNRNPD protein through autophagy‐mediated HNRNPD protein degradation. Given, HNRNPD, one of the best‐characterized ARE‐binding proteins (AUBPs), directly binds to many ARE‐mRNAs and assembles other factors necessary in recruitment of the mRNA degradation machinery, reduced HNRNPD protein leads to less interaction with ARHGDIB mRNA, subsequently increasing ARHGDIB mRNA stability and protein expression, as well as promoting BC cell invasion. Our findings provide significant insight into understanding the nature of the ATG7‐mediated autophagic mechanism implicated in BC invasion.

Autophagy senses stress signals and allows the lysosomal recycling and degradation of cellular components.^22^ Many reports favor the notion that autophagy suppresses tumorigenesis.^23^, ^24^ For example, autophagy plays a tumor suppressive role in the clear cell renal cell carcinoma (CCRCC) development via constitutive degradation of EPAS1.^25^ Mathew et al. report that autophagy reduces DNA damage and chromosomal instability, as well as eliminates SQSTM1 protein to exhibit its tumor suppressor activity.^24^, ^26^ On the other hand, autophagy plays a role in triggering tumor initiation and protecting tumor cells from undergoing apoptosis.^27^ Inhibition of ATG5 has been found to cause antitumor activity in human gastric cancer.^28^ Autophagy has also been reported to promote hepatocellular carcinoma cell invasion through activation of TGF‐βSMAD3‐dependent signaling in regulation of EMT (epithelial–mesenchymal transition).^29^ Here, we have found that autophagy is activated in mouse invasive BCs. And that autophagy is also much more activated in human highly invasive BC cell than that observed in normal UROtsa cells. Moreover, for the first time, we discovered that ATG7‐mediated autophagy plays a crucial role for human BC invasion in vitro and in xenograft tumor growth in vivo, which provides significant insight into understanding ATG7‐mediated autophagy in bladder carcinogenesis and progression.

Autophagy is upregulated in RAS‐transformed cancer cells, in hypoxic tumor regions, and functions in the growth, tumorigenesis, survival, invasion, and metastasis of the transformed cells.^30^ It has recently been reported that deficiency in SQSTM1‐mediated autophagy decreases tumorigenicity of oncogenic KRAS transformed cells by interference of autophagosome cargo delivery or autophagosome formation.^31^ Lung tissue–specific inactivation of ATG5 and its mediated autophagy marked oxidative stress impairs mitochondrial energy homoeostasis and a constitutively active DNA damage response, and also inhibits the progression of KRAS^G12D^‐driven lung cancer.^32^ Lung‐specific ATG7 deficiency induces dysfunctional mitochondria and cell death, and finally reduces lung tumor burden and inhibits KRAS^G12D^‐driven lung tumor growth.^3^ In the current study, ATG7‐dependent autophagy is found to play promotive roles in BC invasion by autophagic removal of the HNRNPD protein, thereby causing an increase in ARHGDIB mRNA stability and protein expression. It is noted in our most recent studies that SESN2‐dependent autophagy is required for anchorage‐independent growth inhibition by the anticancer compound isorhapontigenin (ISO) in human BC T24 and UMUC3 cells.^23^ Although we can't provide the exact mechanisms underlying the diversity effects of autophagy in mediation of anticancer and cancer development, we anticipate that there might be differential mechanisms that can control the divergent outcomes of BC cell growth or inhibition between SESN2‐dependent and ATG7‐dependent autophagy. Further elucidation of this specific question may result in a full understanding of the nature of autophagy in cancer development and anticancer effect; therefore, these studies are currently ongoing in our research group.

Although ATG7‐dependent autophagy has been reported in a variety of cancers, its involvement in human BC invasion has never been explored. Previous studies report that ATG7 downregulation elevates SNAI1 and SNAI2 expression and promotes glioblastoma cell invasion.^33^ However, it also has been reported that the inhibition of ATG7 suppresses tumor growth through a microbiome‐influenced immune response.^34^ ATG7 deletion results in the inhibition of prostate cancer tumor formation in phosphatase and tensin homolog (PTEN) defect mice^35^ and prevents melanoma development due to BRAF^V600E^ and PTEN deletion by increasing oxidative stress and overcoming senescence.^36^ In the current study, we have found that ATG7 is elevated in T24 and UMUC3 cells in comparison to UROtsa cells in vitro and in mouse invasive BCs in vivo. Given that ATG7 upregulation is observed in 9 out of 11 cases from the The Cancer Genome Atlas (TCGA) bladder cancer database (Table S1, Supporting Information), we further tested the relationship between ATG7 and BC invasion in an in vitro cell culture model and tumor growth in xenograft tumor model. The results consistently demonstrate that ATG7 mediates BC cell autophagy, which is also crucial for BC invasion and tumor growth, as demonstrated by the inhibition of BC autophagy. Moreover, we define ARHGDIB as the ATG7 downstream effector responsible for its mediation of BC invasion. Our studies present very strong evidence indicating that ATG7‐dependent autophagy acts as a tumor positive regulator for BC invasion and growth.

ATG7 expression might be regulated at mRNA levels and protein levels. miRNAs are important regulators of gene expression in eukaryotic cells by targeting either mRNA stability or protein translation.^37^ Although miRNAs exert their functions through sequence‐specific binding to mRNA of their target genes, it has been revealed that miRNAs also have cell‐type‐specific signatures on target mRNA expression and are stage‐specific during cancer progression.^38^ MIR17 has been reported to inhibit ATG7 expression, therefore improving the sensitivity of human glioblastoma cells to temozolomide and low‐dose ionizing radiation treatments.^39^ The MIR290‐295 cluster is also reported to suppress ATG7 by directly targeting its 3′‐UTR in melanoma cells.^39^ Our results reveal that ATG7 upregulation in invasive human BC cell lines is only observed in the levels of mRNA and protein, not in the level of ATG7 promoter‐driven transcription, strongly suggesting that the upregulation occurs at mRNA stability. It is well known that miRNA regulates the stability of mRNA by binding to the 3′‐UTR region of its targeted mRNA. Bioinformatics analysis showed that there were multiple putative miRNA binding sites in the ATG7 mRNA 3′‐UTR, as listed in Figure S6B in the Supporting Information. The potential miRNA was evaluated in BC cells by real‐time PCR. MIR190A is showed to be upregulated in highly invasive T24 and UMUC3 cells. The upregulation of MIR190A is also observed in human invasive BCs. Moreover, MIR190A upregulation is also found in 9 out of 11 cases from the TCGA bladder cancer database (Table S1, Supporting Information). Our studies also provide the exclusive evidence supporting MIR190A as a novel critical mediator for the stabilization of ATG7 mRNA in human BCs by using ectopic expression of MIR190A and MIR190A specific antisense in both T24 and UMUC3 cells, as well as the MIR190A binding site mutation in the ATG7 3′‐UTR‐luciferase reporter, which results in the loss of regulatory effects of MIR190A or MIR190A inhibitor on ATG7 3′‐UTR activity. Our results provide evidence of a first‐time link between MIR190A overexpression and BC invasion, which offers new insight into the therapeutic targeting of MIR190A for advanced bladder cancers.

ARHGDIB belongs to a family of RHO guanosine diphosphate dissociation inhibitors (ARHGDIs), and it has been reported that ARHGDIB is a tumor suppressor gene and an aggressive human cancer marker.^40^ In contrast to the tumor suppressive function of ARHGDIB, evidence demonstrates the oncogenic function of ARHGDIB. For example, depletion of ARHGDIB expression inhibits the ability of invasion and migration of pancreatic carcinoma.^10^ ARHGDIB is also reported to promote cell invasion and proliferation by activating Akt in hepatocellular carcinoma.^41^ Here, we show that knockdown of ATG7 almost completely abolished ARHGDIB expression in both T24 and UMUC3 cells, as well as in their xenograft tumors. Our unpublished studies demonstrate that ARHGDIB expression is upregulated in mouse invasive BCs; by using gain‐ and loss‐function studies, we show that ARHGDIB promotes human BC lung metastasis (Jin H and Huang C, et. al, data not shown). It is highly significant to observe that simple ectopic expression of ARHGDIB completely restored the invasive ability in invasion‐deficient UMUC3(shATG7) cells. These results suggest that ARHGDIB is an ATG7 downstream effector responsible for its positive regulation of BC invasion. Moreover, we have identified that ATG7 stabilizes ARHGDIB mRNA through its autophagic inductive activity, which further degrades HNRNPD protein, while HNRNPD is an ARHGDIB mRNA binding protein.

In summary, ATG7 and its mediated autophagy are first found to be upregulated in mouse invasive BCs, human invasive BC cell lines, and human BC tissues, which play important roles in BC invasion. Further, we have discovered that MIR190A is an ATG7 upstream positive regulator for ATG7 overexpression, whereas ARHGDIB is the ATG7 downstream effector and is responsible for promoting BC invasion. Moreover, ATG7 promotes ARHGDIB mRNA stability through its autophagic degradation of HNRNPD protein. Our new findings raise the potential for developing an ATG7/autophagy‐based‐specific therapeutic strategy for treatment of human BC patients.

## 4 Experimental Section

*Plasmids, Reagents, and Antibodies*: The plasmid of the human ATG7 promoter (from −1398 to −227)‐driven luciferase reporter was constructed with KpnI and BglII using genomic DNA purified from UMUC3 cells (Figure S5, Supporting Information). Human ATG7 3′‐UTR luciferase reporter has already been described (Figure S6A, Supporting Information).^39^ ATG7 3′‐UTR mutant luciferase reporter (MIR190A binding site was mutated) was cloned into the pMIR luciferase reporter. V5‐DEST‐MIR190A and its control construct were kindly gifted by Dr. Ping‐Yee Law (Department of Pharmacology, University of Minnesota, Minneapolis, MN, USA). The plasmid of ARHGDIB promoter‐driven luciferase reporter was synthesized by Cyagen Bioscience and was inserted into pGL3 Basic plasmid. The GFP‐tagged ARHGDIB was kindly gifted by Dr. Martin A. Schwartz (Robert M. Berne Cardiovascular Research Center, University of Virginia, Charlottesville, Virginia).^42^ The Myc‐ATG7 construct was obtained from Addgene (Cambridge, MA). The GFP‐ATG7 plasmid was constructed with KpnI and NotI using cDNA purified from UMUC3 cells. MIR196B constitutively expressed plasmid was kindly gifted from Dr. Wen‐Chang Lin (Institute of Biomedical Sciences, Academic Sinica, Nankang, Taipei 115, Taiwan, Republic of China).^43^ The HA‐tagged HNRNPD (38066) and ATG7 constitutively expressed plasmid (24921) were obtained from Addgene (Cambridge, MA, USA). The plasmids of short hairpin RNA specifically targeting ATG7 (shATG7) (human, RHS4531‐EG10533), HNRNPD (shHNRNPD) (human, RHS4531‐EG3184), and the plasmid of antisense of MIR190A (HmiR‐AN0258‐AM03) were purchased from GeneCopoeia (Rockville, MD, USA). All plasmids were prepared by the Plasmid Preparation/Extraction Maxi Kit (10063) from Qiagen (Valencia, CA, USA). BBN (B0938) was purchased from TCI AMERICAN (Cambridge, MA, USA). The chemicals Cycloheximide (CHX) (239763) and Act D (129935) were purchased from Calbiochem (Billerica, MA, USA). Bafilomycin A1 (sc‐201550A) was bought from Santa Cruz (St. Louis, MO, USA). The antibodies of GAPDH (5174S), RHOA (2117S), RAC1,2,3 (2465S), GFP (2956S), ELAVL1 (12582S), and Autophagy Antibody Sample Kit (4445S) were purchased from Cell Signaling Technology (Beverly, MA, USA). The antibody specific against HNRNPD (OAAF01114) was bought from Aviva Systems Biology (San Diego, CA, USA). Antibodies against NCL (sc‐13057), ARHGDIA (sc‐6047), and ARHGDIB (sc‐32227) were bought from Santa Cruz (Dallas, Texas 75220 USA), while antibodies against β‐Actin (A5316) were purchased from Sigma (St. Louis, MO, USA).

*Cell Lines and Cell Culture*: UROtsa, UMUC3, and T24 cells were used in the previous studies.^44^ Before/after utilization for research, PowerPlex 16 HS System was used to do DNA tests and authenticate all the cell lines by Genetica DNA Laboratories (Burlington, NC, USA).

*Western Blot Analysis*: The whole BC cell extracts were prepared and treated as described in the previous studies.^45^ The images were acquired by scanning with the phosphor imager (Typhoon FLA 7000, GE, Pittsburgh, PA).

*Transfection and Luciferase Assay*: In Vitro Transfection Reagent PolyJet DNA (SL100468) (SignaGen Laboratories, Rockville, MD, USA) was used and described in the previous studies.^45^ The luciferase reporters and pRL‐TK were transiently transfected into the indicated cells. 24 h after transfection, the luciferase Assay System kit (E1960) (Promega, Madison, WI, USA) was used to determine the luciferase activity. The results were normalized by internal TK signal. All experiments were done in triplicate and the results expressed as mean ± standard error.

*Reverse Transcription‐Polymerase Chain Reaction (RT‐PCR)*: Total RNA was extracted using the TRIzol reagent (15596026) (Invitrogen, Grand Island, NY, USA), and then. 5.0 µg RNA was used for first‐strand cDNA synthesis with oligo (dT) 20 primer by using Super‐Script First‐Strand Synthesis system (18080051) (Invitrogen, Grand Island, NY, USA). The PCR product was analyzed by agarose gel. The densitometric analyses of the product bands were performed using the Image Quant 5.2 software (GE Healthcare, Pittsburgh, PA, USA). The primers used in this study were: human ATG7 (Forward, 5′‐GCC AAG ATC TCC TAC TCC AATC‐3′; Reverse, 5′‐CAG AAG TAG CAG CCA AGC TTGT‐3′) and human β‐Actin (Forward, 5′‐CTC CAT CCT GGC CTC GCT GT‐3′; Reverse, 5′‐GCT GTC ACC TTC ACC GTT CC‐3′).

*Quantitative RT‐PCR for mRNA and miRNA Assay*: Fast SYBR Green Master Mix kit (Applied Biosystems, 4385614) was used to conduct real‐time PCR in the 7900HT Fast Real‐Time PCR System (Applied Biosystems, Foster City, CA, USA). The primers used were: human ARHGDIB (Forward, 5′‐ACC CGG CTC ACC CTG GTT TGT‐3′; Reverse, 5′‐ACC CCA GTC CTG TAG GTG TGC TG‐3′); human HNRNPD (Forward, 5′‐AAA TTG AAT GGG AAG GTG AT‐3′; Reverse, 5′‐GAA CCC ACG CCT CTT ATT G‐3′), and human β‐Actin (Forward, 5′‐CTC CAT CCT GGC CTC GCT GT‐3′; Reverse, 5′‐GCT GTC ACC TTC ACC GTT CC‐3′). The primer for MIR190A (MS00011333) was purchased from Qiagen (Germantown, MD, USA).

*RNA Immunoprecipitation (RNA‐IP) Assay*: 293T cells were transiently transfected with HA‐HNRNPD. Then, the RNA‐IP assay was performed as described in the studies previously.^46^ RT‐PCR was performed to identify the mRNA presented in the immune‐complex.

*Cell Migration and Invasion Assay*: Control inserts without matrigel and permeable support for 24‐well plate with 8.0 µm transparent PET membrane were purchased from Corning Incorporated (Corning, NY, USA) (353097), and the invasion kit (354480) was purchased from BD Biosciences (Bedford, MA, USA). The cell migration and invasion activity of the indicated cells were evaluated as described in the previous studies.^47^ The images of the results were taken under a microscopy, Olympus DP71 (Olympus America Inc. Center Valley, PA, USA), and the number of the cells in each image was counted by the software “Image J.” The invasion rate was normalized with the insert control according to the manufacturer's instruction.

*The Construct of Human ATG7 Promoter‐Driven Luciferase Reporter, ATG7 mRNA 3′‐UTR Mutant Luciferase Reporter, and pLentiIII‐GFP‐ATG7 Plasmid*: The plasmid of ATG7 promoter (from −1398 to −227)‐driven luciferase reporter was constructed by amplifying from genomic DNA isolated from UMUC3 cells based on the NCBI database, using primers: forward, 5′‐ACT GGT ACC ACT GAC ACA CAC AAC CCC CTA CTG AG‐3′ and Reverse, 5′‐ACT AGA TCT GAG AGG CGG CAT CAA ACG CAG CAC A‐3′, and then subcloned into the KpnI and BglII sites of the PGL3‐basic vector, thus originating the ATG7 promoter‐driven luciferase reporter plasmid. The relevant sequence of human ATG7 promoter (from −1398 to −227) was indicated in Figure S5 in the Supporting Information. The seed region of putative MIR190A/ATG7 interacting sequence (see Figure 2P) was introduced with a three‐point mutation to produce the pMIR‐ATG7 3′‐UTR mutant plasmid by using primers: MIRMUTFOR, 5′‐AAT TAA ATA GCT ACA GCG CAT TAA CAA ATT AAT GTT C‐3′ and MIRMUTREV, 5′‐GAA CAT TAA TTT GTT AAT GCG CTG TAG CTA TTT AAT T ‐3′. The plasmid of pLentiIII‐GFP‐ATG7 was constructed by amplifying from cDNA isolated from UMUC3 cells, using primers: forward, 5′‐ CGG GGT ACC CAA GAA ATA ATG GCG GCA G‐3′ and Reverse, 5′‐ ATA AGA ATG CGG CCG CAT CTC AGA TGG TCT CAT C‐3′, and then subcloned into the KpnI and NotI sites of the pLentiIII‐GFP vector, thus originating the pLentiIII‐GFP‐ATG7 plasmid. Constructs were all sequence verified by GENEWIZ (South Plainfield, NJ, USA).

*Human Bladder Cancer Tissue Specimens*: All human studies were performed in compliance with the relevant laws and institutional guidelines, and were approved by the Ethics Committee of Wenzhou Medical University (March 6, 2016). Informed consent was obtained from all patients before sample collection. All human bladder cancer tissue specimens were obtained from patients who underwent radical cystectomy at the Department of Urology of the Union Hospital of Tongji Medical College (Wuhan, China) during 2012 and 2013 and the First Affiliated Hospital of Wenzhou Medical University (Wenzhou, China) between 2015 and 2016, which have been described in the previous study.^48^

*Tumor Xenografts and In vivo BBN Treatment of Mice*: In accordance with NIH guidelines, all animal procedures were approved by the New York University Committee on Animal Resources. The tumor xenograft studies were completed in the Animal Institute of Wenzhou Medical University, which is approved by the Medical Experimental Animal Care Commission of Wenzhou Medical University. The experiment of mice treated with BBN in vivo and the tumor xenograft studies were described in the previous study.^44^ None of the mice were sacrificed or died before the end of the experiment.

*Immunohistochemistry Paraffin (IHC‐P) of Mouse and Human Bladder Specimens*: Mouse or human bladder cancer specimens were formalin‐fixed and paraffin‐embedded. For IHC staining, antibodies specific were used against ATG7 (sc‐33211) (Santa Cruz St. Louis, MO, USA), NCL (sc‐17826) (Santa Cruz St. Louis, MO, USA), HNRNPD (OAAF01114) (Aviva Systems Biology, San Diego, USA), or ARHGDIB (sc‐32227) (Santa Cruz St. Louis, MO, USA). The AxioVision Rel.4.6 computerized image analysis system (Carl Zeiss, Oberkochen, Germany) was used to get the resultant immunostaining images. Image‐Pro Plus version 6.0 (Media Cybernetics, MD, USA) was used to analyze the protein expression by calculating the integrated optical density per stained area (IOD/area).

*Statistical Methods*: Student's *t‐*test was utilized to determine the significance of differences between different groups. The differences were considered to be significant at *p* < 0.05.

## Conflict of Interest

The authors declare no conflict of interest.

## Supporting information

Supplementary
