# ATG7 Promotes Bladder Cancer Invasion via Autophagy‐Mediated Increased ARHGDIB mRNA Stability

**DOI:** 10.1002/advs.202104365

**Published:** 2021-11-17

**Authors:** Junlan Zhu, Zhongxian Tian, Yang Li, Xiaohui Hua, Dongyun Zhang, Jingxia Li, Honglei Jin, Jiheng Xu, Wei Chen, Beifang Niu, Xue‐Ru Wu, Sergio Comincini, Haishan Huang, Chuanshu Huang

*Adv. Sci*. **2019**, *6*, 1801927

https://doi.org/10.1002/advs.201801927

In the originally published article, there are errors found in Figures 3 and 4 panels: the images representing the Transwell of T24(shATG7#2) in Figure 3G, and the Invasion of UMUC3(pLKO.1) and both images of UMUC3(LacZ) in Figure 4A are incorrect. Additionally, the image of the Transwell of shBECN1#1 in Figure S1C (Supporting Information) has been wrongly placed.

**Figure 3:**
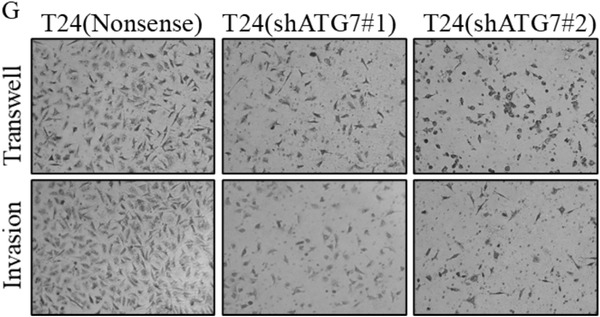
ATG7 overexpression and its mediated autophagy were crucial for BC invasion. G) The invasion abilities of T24(shATG7) and their nonsense transfectants were determined, the migrated and invasive cells were photographed under an Olympus DP71.

**Figure 4:**
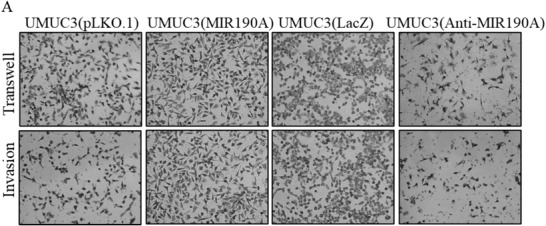
ATG7 was an MIR190A downstream effector responsible for BC invasion. A) The invasion abilities of the indicated cells were evaluated.

**Figure S1:**
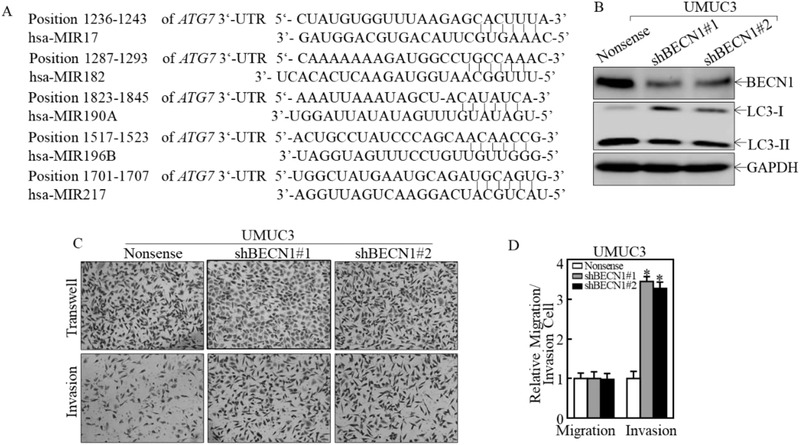
(A) The potential microRNAs binding sites in ATG7 mRNA 3′‐UTR were analyzed using the TargetScan, PicTar, and miRanda databases. (B) BECN1 knockdown constructs were stably transfected into UMUC3 cells. The knockdown efficiency of BECN1 protein and autophagy activity were assessed by Western Blotting. (C) The invasion abilities of BECN1 knockdown in UMUC3 cells were evaluated in comparison to their vector transfectants using a BD BioCoatTM MatrigelTM Invasion Chamber applied with the matrigel. Following incubation for 24 h, the cells were fixed and stained, as described in “Materials and Methods”. The migrated and invasive cells were photographed with an Olympus DP71 and the number of the cells was calculated by the software “Image J”. (D) The invasion rate was normalized with the insert control according to the manufacturer’s instruction. The results are presented with the mean±SD from triplicate. Student’s t‐test was utilized to determine the p‐value, *p < 0.05.

The correct figures panels are presented below. The corrections do not affect the results or conclusions of this work. The authors apologize for any inconvenience this may have caused.

## Supporting information

Supporting Information

