# Supplementary material for: ATG7 Promotes Bladder Cancer Invasion via Autophagy‐Mediated Increased ARHGDIB mRNA Stability
Source: Adv Sci (Weinh). 2019 Feb 22;6(8):1801927. doi: 10.1002/advs.201801927 (PMC6468970; doi:10.1002/advs.201801927)
Supplement: Supplementary file 1 — Supplementary [file ADVS-6-1801927-s001.pdf]

Copyright WILEY-VCH Verlag GmbH & Co. KGaA, 69469 Weinheim, Germany, 2019.

Supporting Information

for *Adv. Sci.,* DOI: 10.1002/advs.201801927

ATG7 Promotes Bladder Cancer Invasion via Autophagy-Mediated Increased ARHGDIB mRNA Stability

Junlan Zhu, Zhongxian Tian, Yang Li, Xiaohui Hua, Dongyun Zhang, Jingxia Li, Honglei Jin, Jiheng Xu, Wei Chen, Beifang Niu, Xue-Ru Wu, Sergio Comincini, Haishan Huang, and Chuanshu Huang**

Supplementary Figure. 1

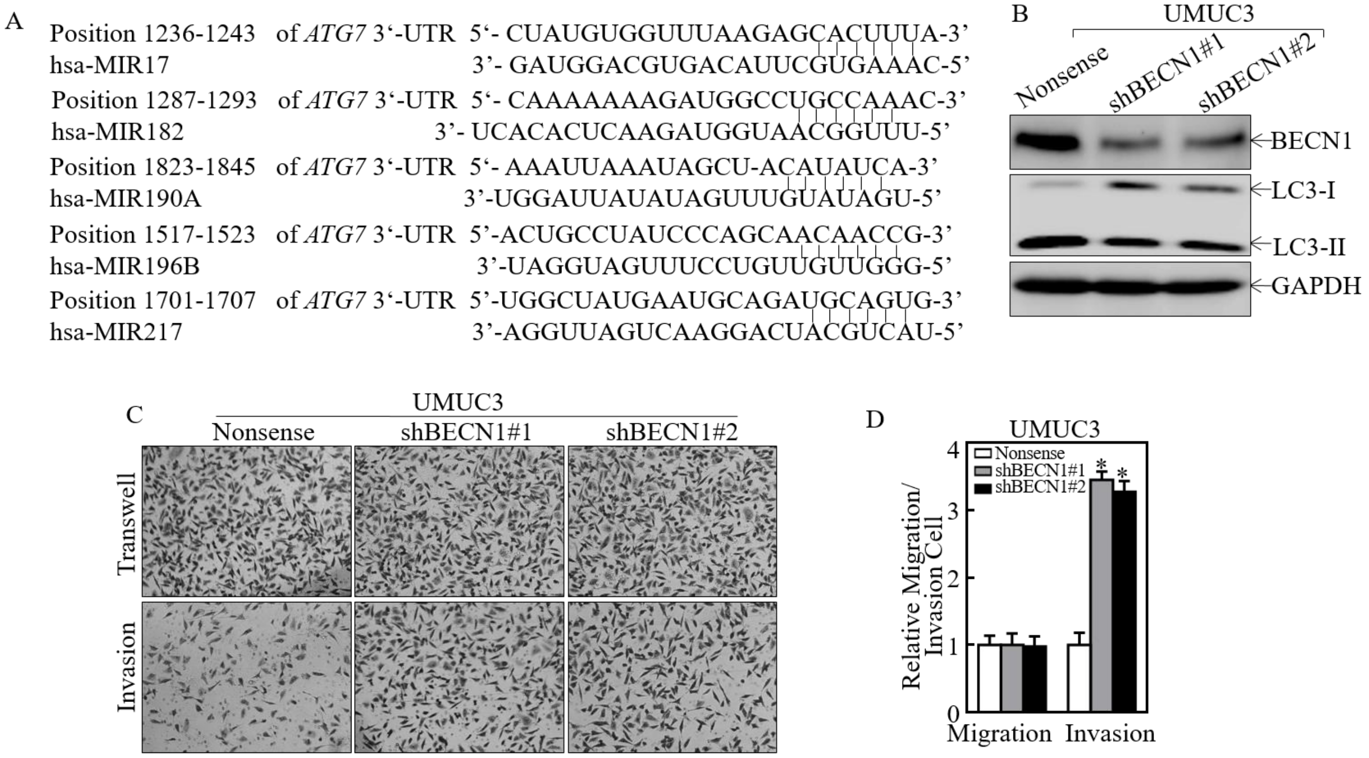

Figure. S1 (A) The potential microRNAs binding sites in ATG7 mRNA 3'-UTR were analyzed using the TargetScan, PicTar, and miRanda databases. (B) BECN1 knockdown constructs were stably transfected into UMUC3 cells. The knockdown efficiency of BECN1 protein and autophagy activity were assessed by Western Blotting. (C) The invasion abilities of BECN1 knockdown in UMUC3 cells were evaluated in comparison to their vector transfectants using a BD BioCoat™ Matrigel™ Invasion Chamber applied with the matrigel. Following incubation for 24 h, the cells were fixed and stained, as described in "Materials and Methods". The migrated and invasive cells were photographed with an Olympus DP71 and the number of the cells was calculated by the software "Image J". (D) The invasion rate was normalized with the insert control according to the manufacturer's instruction. The results are presented with the mean±SD from triplicate. Student's t-test was utilized to determine the p-value, $*p < 0.05$.

Supplementary Figure. 2

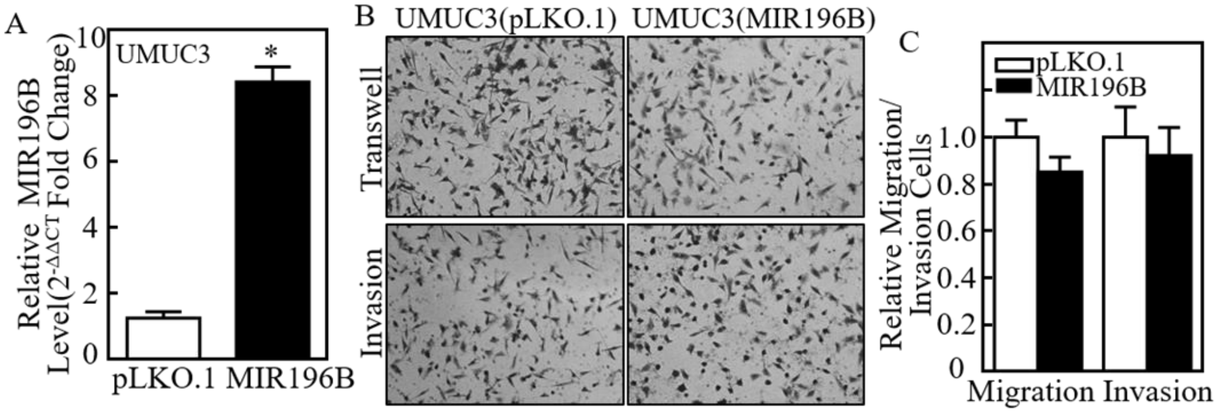

Figure. S2 (A) MIR196B constitutively expressed plasmid was stably transfected into UMUC3 cells. The transfectants were identified by Real-time PCR. Bars represent mean±SD from three independent experiments. The asterisk (*) indicates a significant increase in comparison to UMUC3(pLKO.1) control cells. Student's t-test was utilized to determine the p-value, $^{*}p < 0.05$. (B) The invasion abilities of

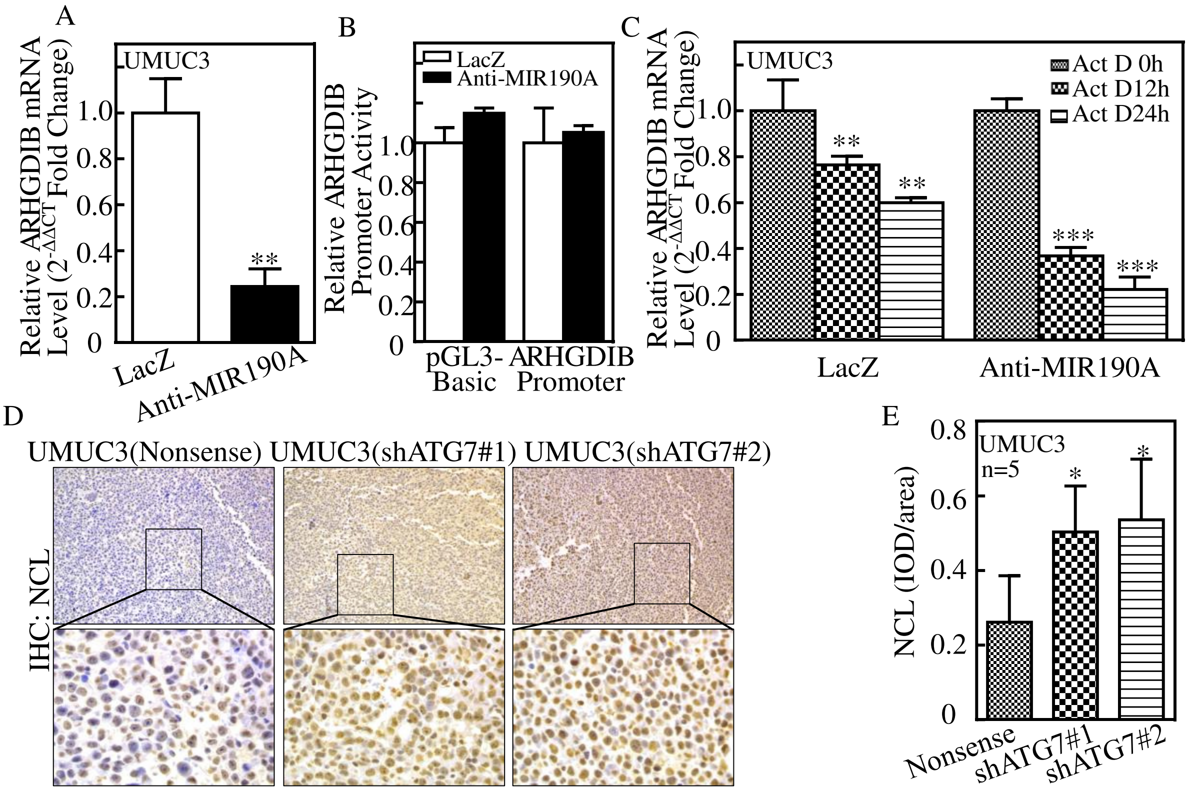

MIR196B transfectants in UMUC3 cells were evaluated in comparison to their vector transfectants using a BD BioCoatTM MatrigelTM Invasion Chamber applied with the matrigel. Following incubation for 24 h, the cells were fixed and stained, as described in "Materials and Methods". The migrated and invasive cells were photographed with an Olympus DP71 and the number of the cells was calculated by the software "Image J". (C) The invasion rate was normalized with the insert control according to the manufacturer's instruction. The bars represent mean±SD from three independent experiments. Student's t-test was utilized to determine the p-value, $p > 0.05$.

Supplementary Figure. 3

Figure. S3 (A) UMUC3(LacZ) and UMUC3(Anti-MIR190A) cells were cultured in 6-well plates till cell density reached 80–90% and the cells were then extracted for total RNA with Trizol reagent. Real-time PCR was used to determine ARHGDIB mRNA expression and ACTB was used as an internal control. Bars represent mean±SD from three independent experiments. Student's t-test was utilized to determine the p-value, **p < 0.05. (B) Human ARHGDIB promoter-driven luciferase activity was evaluated in the indicated cells. The results were normalized by internal TK activity. Bars represent mean±SD from three independent experiments. Student's t-test was utilized to determine the p-value, p > 0.05. (C) UMUC3(LacZ) and UMUC3(Anti-MIR190A) cells were seeded into 6-well plates. After synchronization, the cells were used to determine ARHGDIB mRNA stability in the presence of Actinomycin D (Act D) using Real-time PCR. ACTB was used as an internal control. Bars represent mean±SD from three independent experiments. Student's t-test was utilized to determine the p-value **p < 0.05. (D & E) HE staining and immunohistochemistry staining were performed to evaluate NCL expression in BBN-induced mouse invasive BCs. The IHC images were captured using the AxioVision Rel.4.6 computerized image system and the protein expression levels were analyzed. Bars represent mean±SD from 5 mice in each group. Student's t-test was utilized to determine the p-value, *p < 0.05.

Supplementary Figure. 4

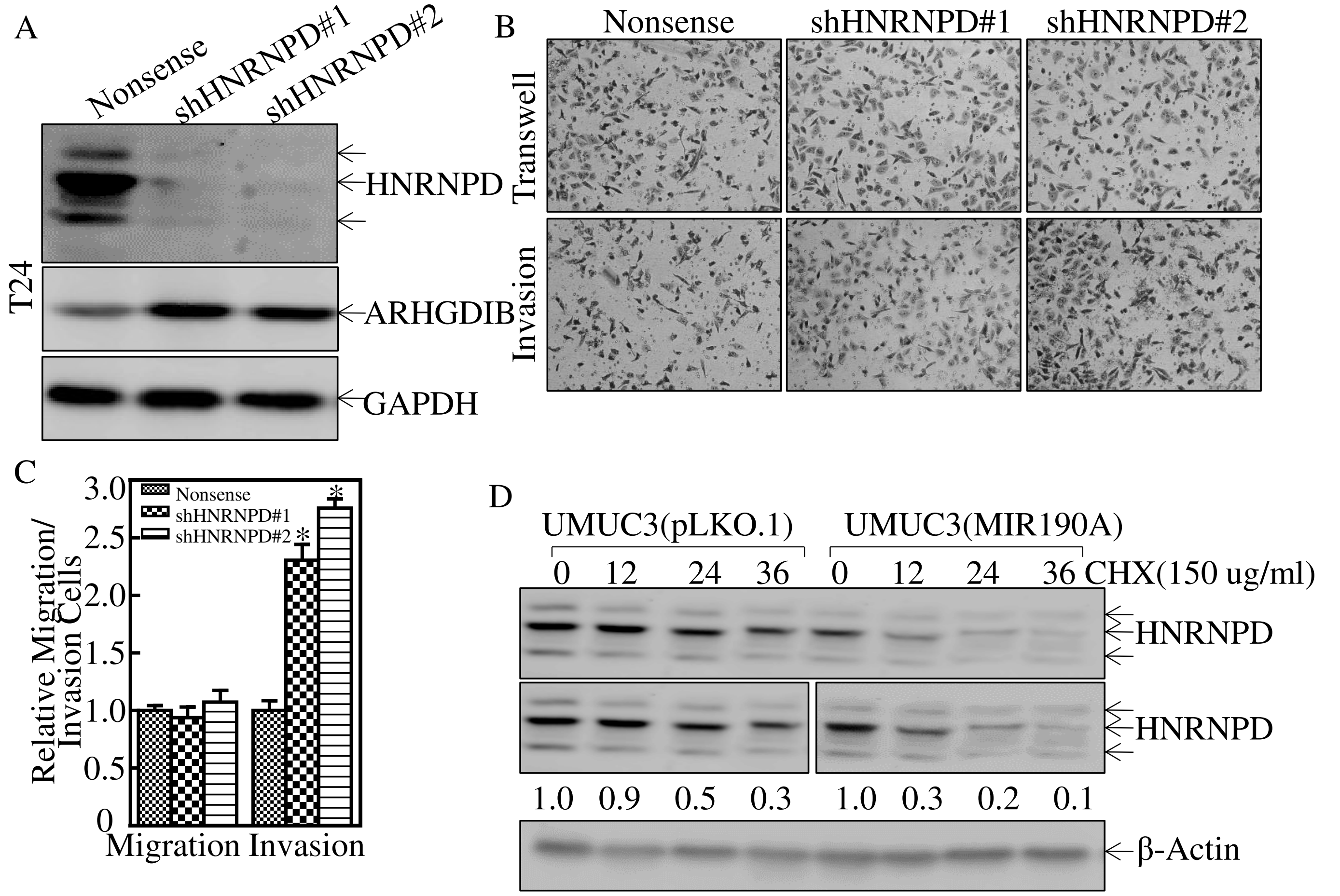

Figure. S4 (A) HNRNPD knockdown constructs were stably transfected into T24 cells, the transfectants were identified by Western Blotting, the cells then were used to determine their invasive abilities in comparison to the T24(nonsense) control transfectants. After incubation for 24 h, the cells were fixed and stained. The migrated and invasive cells were photographed with an Olympus DP71. The number of the cells was calculated by the software "Image J" (B). (C) The invasion rate was normalized with the insert control according to the manufacturer's instruction. The results are presented with the mean±SD from

Human ATG7 Promoter (-1398---227)

5'-
TCATCCCACTTCCCATATTCGGTTGTTTCCAGCCTTGTTTACCTGCCTTTGGGGAGGGTGGGGGG
TGGGGGAACCCTCTTTTTCCCTAACCATTGAAACATCTGCGGATGTTTTGGTCAGAGTGATCTC
ACTATTGCAATAGTCTCCCTCTGCTATTGCACGGTTCCTTCTCTCCCACCTCTCTCACTTCTTGCA
GTAATCCTTTCTTTTTTTTTTTTTTGAGACGGAGTCTCGCTCTGTCGCCCAGGCTGGAGTGCAG
TGGTGGGATCTCGGCTCACTGCAAGCTCCGCCTCCCGGGTTCACGCCATTCTCCTGCCTCAGCC
TCCCAAGTAGCTGGGACTACAGGCGCCCGCCACCACGCCCGGCTAATTTTTTTGTATTTTTAGTA
GAGACGGGGTTTCACCGTTTTAGCCGGGATGGTCTCGATCTCCTGACCTCGTGATCCGCCCGCC
TCGGCCTCCCAAAGTGCTGTGATTACAGGCGTGAGCCACCGCGCCGGGCCTGCAGTAATCCTT
TCTAATAAGTATCCTTATTAGTCTGGATTTGTTTTCTTATTTGCCAGAATCAAACAAACTTCAGAT
TACGTTCCTGGCCCAAAAGCCCTTTAACTTTCAGCAAGCCACCTAACTTTTCTTAGATTCTGTTC
TTTCATATATAACTTGGGGATATGTTTCAGGACTCTTGTGAGGATTAGAGATGTAAAACCCTGAA
AACATGGTGGCTAGTTAATTAATAGCAGTCATCGCTCTTGTTGTTATGAGTCTAGAGTGGTATATC
AGAGTTAATTTGTGAAAGGCCTTACAGGCCAGACAGAGAAACTTGTCATAATCACTGCTGTATC
TTCAACACCTGTCCAGTGCTTTGCACGCGCAGAGTATCTAACCAAGTATTGAGAGGGAATAACT
TTATCTCACTGACAGTGAGGAGCTGTGAGAATGATGGCTGGTTTAGAAAAGGCAGGTTGGTCA
CTGTCGACGTTCACTGGCCTTTTCCTACTAAAATTCTCATCTCCTGGCTCTCCACACCTGCCACC
CTGATGGCCCCTGTGCTGCGTTTGATGCCGCCTCTCCTGGAGAATGACCATGGTGATCATTCCT
GTCATCCTCTGAAATCAAAAGAGAGAACGTGGGCACTTTCTTAAAAGCCTGAAGGGAATGTAG
ACATTCCGACA-3'

triplicate. Student's t-test was utilized to determine the p-value, *$p < 0.05$. (D) HNRNPD protein stabilities were evaluated in presence of Cycloheximide (CHX), for the indicated times, in UMUC3(pLKO.1) and UMUC3(MIR190A) cells.

Supplementary Figure. 5

Figure. S5 The sequence of human ATG7 promoter from -1398 to -227.

Supplementary Figure. 6

A

Human ATG7 3'-UTR(1146--2126)

5'-
CATGGGGACACAGCCGGCACAGGTGCAGGGCCCGAGTCCGCCCACCCAGCCTGGCGCTGA
AACTGCACACGTACACTATGTGGTTTAAGAGCACTTTATTATTGTTCTTAAGGCTACTTTTAAG
TACAAAAAAAGATGGCCTGCCAAACCTTTTTTTTTCTTCTTCCAGGAAAAACAGGCCACAGA
GAATGGTATATTACAGATTTACACACATGAAGAGAAGGTCAGAGCGCACTGCAGGCAGCGCG
GCTCTGGGAAGAACTTCACGGAGCCCCTTCTTAGAGCAGGGAGGGGGCTTTCTCAGTGAAA
TGTTTGGTTTTCTGCTGCCTCCTCTGCCCCAGGCCCCCCTCCAGGGTACTGCCTATCCCAGAT
AGGTCAGTGCACCAGGGACCCGGCCGCCAGCACCGCCGACCCCTCCCAGAGTGACGCCCTT
GTTCACTGACAAAGAGACCTGTCCCAGGAGTGTCCTCCACCGAGCCGGTCAGCTGTGGGTG
GTTTTCCTGTTACGACGCTCAGTAGCCTGTAGCAATAACAAACTCGTGGCTATGAATGCAGAT
GCAGTGTTCTCATAGAATAACTGTTCCTGCACTTTTACAGACAAATCTACGACAAAAAAAAA
GATCAACTTTTTTTTTCCGAACAACAAAAAAAAATGAATGATTACAATAGGAAAGGGAAAAAT
TAAATAGCTACATATCATTAACAAATTAATGTTCTTCAAAAAATACCTACAAATTTCTCTGTAC
ATTCTTTACGCACAGCGTAACGATGGTCTCAAAATCACCCATATAGAAAAGTGTTCTCAACGA
TTTTTCCTACAGAAAATATAGGGGCCTGAATGCCAAAGCTTGGAAGCCCAGTACAGTGGGAG
TGAAATGTGTGCGGGGCAAGGAGAAGGGCTTTTCTTTCCTCCACTTTTCAAAGGCCTGCAGC
CACTCTGTGACTACAAGAGCCAGTCCTCCGACCTTTTCACCCAGTGCCA-3'

B

miRNA families broadly conserved among vertebrates for binding to human ATG7 3'-UTR(1146--2126) are listed: MIR17-5p; MIR20-5p; MIR93-5p; MIR106-5p; MIR519-3p; MIR142-5p; MIR302-3p; MIR372-3p; MIR373-3p; MIR520-3p; MIR129-5p; MIR192-5p; MIR215-5p; MIR196B-5p; MIR1271-5p; MIR182-5p; MIR7-5p; MIR137; MIR204-5p; MIR211-5p; MIR190A-5p; MIR210-5p; MIR217-5p; MIR141-3p; MIR200-5p; MIR221-3p; MIR146-5p; MIR96-5p; MIR129-5p; MIR202-5p.

Figure. S6 (A) The sequence of human ATG7 3'-UTR from 1146 to 2126. (B) The potential microRNA families broadly conserved among vertebrates for directly binding to human ATG7 3'-UTR from 1146 to 2126 were analyzed by the TargetScan, PicTar, and miRanda.

Supplementary Table 1

Table S1. Available information on the co-relation of expression of ATG7 and MIR190A in the TCGA bladder cancer database.

	normal-MIR190A	tumor-MIR190A	Fold change	normal-ATG7	tumor-ATG7	Fold change	vital status	days to death	age at initial pathologic diagnosis	gender
TCGA-GD-A2C5	3.051465	3.153024	1.033282	7.268675	8.111414	1.115941	alive	NA	53	female
TCGA-BT-A2LA	2.946229	5.952318	2.020317	9.970506	8.153989	0.817811	alive	NA	54	male
TCGA-CU-A0YN	1.13953	1.177628	1.033433	4.126282	8.085731	1.959568	dead	393	60	male
TCGA-BL-A13J	0.798138	0.998694	1.25128	4.700431	8.174075	1.739006	dead	81	65	male
TCGA-CU-A0YR	1.520341	2.415732	1.588941	6.5207	17.50831	2.685036	dead	460	83	male
TCGA-BT-A2LB	3.861158	8.523862	2.207592	5.420452	11.97825	2.209826	dead	492	73	female
TCGA-BT-A20N	0.771078	4.294505	5.569482	5.010572	8.526783	1.701758	dead	795	72	male
TCGA-BT-A20W	0.564775	4.709537	8.338784	8.561534	10.76339	1.257181	dead	254	71	male
TCGA-BT-A20U	0.619989	5.305531	8.55746	4.919076	8.846952	1.798499	dead	455	70	female
TCGA-BT-A20Q	0.1561	1.699947	10.89012	3.342783	8.447315	2.527031	dead	593	73	male
TCGA-BT-A20R	0.267185	4.033177	15.09507	4.248706	9.13639	2.150394	dead	154	79	female
