# Supplementary material for: ATG7 Promotes Bladder Cancer Invasion via Autophagy‐Mediated Increased ARHGDIB mRNA Stability
Source: Adv Sci (Weinh). 2021 Nov 17;8(22):2104365. doi: 10.1002/advs.202104365 (PMC8596123; doi:10.1002/advs.202104365)
Supplement: Supplementary file 1 — Supporting Information [file ADVS-8-2104365-s001.docx]

**Figure. S1** (A) The potential microRNAs binding sites in ATG7 mRNA 3’-UTR were analyzed using the TargetScan, PicTar, and miRanda databases. (B) BECN1 knockdown constructs were stably transfected into UMUC3 cells. The knockdown efficiency of BECN1 protein and autophagy activity were assessed by Western Blotting. (C) The invasion abilities of BECN1 knockdown in UMUC3 cells were evaluated in comparison to their vector transfectants using a BD BioCoatTM MatrigelTM Invasion Chamber applied with the matrigel. Following incubation for 24 h, the cells were fixed and stained, as described in “Materials and Methods”. The migrated and invasive cells were photographed with an Olympus DP71 and the number of the cells was calculated by the software “Image J”. (D) The invasion rate was normalized with the insert control according to the manufacturer’s instruction. The results are presented with the mean±SD from triplicate. Student’s t-test was utilized to determine the p-value, *p < 0.05.
